# Structural and functional changes of the visual cortex in early Huntington's disease

**DOI:** 10.1002/hbm.24322

**Published:** 2018-08-24

**Authors:** Emma M. Coppen, Jeroen van der Grond, Anne Hafkemeijer, Jurriaan J. H. Barkey Wolf, Raymund A. C. Roos

**Affiliations:** ^1^ Department of Neurology Leiden University Medical Center Leiden the Netherlands; ^2^ Department of Radiology Leiden University Medical Center Leiden the Netherlands; ^3^ Department of Methodology and Statistics, Institute of Psychology Leiden University Leiden the Netherlands; ^4^ Leiden Institute for Brain and Cognition Leiden University Leiden the Netherlands; ^5^ Department of Molecular Epidemiology Leiden University Medical Center Leiden the Netherlands

**Keywords:** brain atrophy, functional MRI, Huntington's disease, structural MRI, visual cognition, visual cortex

## Abstract

Huntington's disease (HD) is an autosomal‐dominant inherited neurodegenerative disorder characterized by motor disturbances, psychiatric disturbances, and cognitive impairment. Visual cognitive deficits and atrophy of the posterior cerebral cortex are additionally present in early disease stages. This study aimed to assess the extent of structural and functional brain alterations of the visual cortex in HD gene carriers using different neuroimaging modalities. Structural and functional magnetic resonance imaging data were acquired from 18 healthy controls, 21 premanifest, and 20 manifest HD gene carriers. Voxel‐based morphometry (VBM) analysis and cortical thickness measurements were performed to assess structural changes in the visual cortex. Brain function was measured by assessing neuronal connectivity changes in response to visual stimulation and at rest in visual resting‐state networks. Multiple linear regression analyses were performed to examine the relationship between visual cognitive function and structural imaging measures. Compared to controls, pronounced atrophy and decreased neuronal function at rest were present in associative visual cortices in manifest HD. The primary visual cortex did not show group differences in cortical thickness and in vascular activity after visual stimulation. Thinning of the associative visual cortex was related to worse visual perceptual function. Premanifest HD gene carriers did not show any differences in brain structure or function compared to controls. This study improves the knowledge on posterior brain changes in HD, as our findings suggest that the primary visual cortex remains preserved, both structurally and functionally, while atrophy of associative visual cortices is present in early HD and linked to clinical visual deficits.

## 1 INTRODUCTION

Visual processing is involved in routine daily functioning, such as walking, driving a car, and in social communication. In the human brain, transmission of incoming stimuli from the retina pass the afferent visual pathway via the optic nerve, optic tract, lateral geniculate nucleus in the thalamus, and optic radiation to the primary visual (striate) cortex in the occipital lobe and higher posterior cortical areas (Prasad & Galetta, 2011). Higher level visual processing occurs in the associative extra‐striate regions surrounding the primary visual cortex, from the secondary visual cortex to the ventral occipital‐temporal pathway and dorsal occipital‐parietal pathway (Ungerleider & Haxby, 1994). In Huntington's disease (HD), an autosomal‐dominant inherited neurodegenerative disorder, progressive structural changes in the posterior cerebral cortex can be detected in early stages of the disease, while frontal and temporal regions remain less affected (Johnson et al., 2015; Nana, Kim, Thu, Oorschot, & Tippett, 2014; Nopoulos et al., 2010; Rosas et al., 2008; Rüb et al., 2015; Tabrizi et al., 2009). The manifest stage of HD is clinically characterized by progressive motor disturbances such as chorea and dystonia, cognitive decline, and psychiatric symptoms (Bates et al., 2015; Roos, 2010). Cognitive decline mainly involves progressive impairment in executive function (Dumas, van den Bogaard, Middelkoop, & Roos, 2013). Nonetheless, neuropsychological studies assessing visual cognitive function in HD reported impairments in several visual domains, specifically tasks involving visual object perception (Gómez‐Tortosa, del Barrio, Barroso, & García Ruiz, 1996; Lemiere, Decruyenaere, Evers‐Kiebooms, Vandenbussche, & Dom, 2004), facial emotion recognition (Bora, Velakoulis, & Walterfang, 2016; Kordsachia, Labuschagne, & Stout, 2017), visuospatial processing, and visual working memory (E. Dumas et al., 2012; Johnson et al., 2015; Labuschagne et al., 2016).

Striatal atrophy is considered to be the origin of the characteristic choreatic motor signs, but it is suggested that other symptoms of HD might be related to cortical degeneration (Nana et al., 2014; Thu et al., 2010; Waldvogel, Kim, Thu, Tippett, & Faull, 2012). Thinning of regions in the occipital lobe has been linked to worse performance on cognitive visuospatial tasks, which implies a distinct association between higher level cognitive performance and occipital degeneration in HD gene carriers (Johnson et al., 2015; Rosas et al., 2008).

In contrast to many structural magnetic resonance imaging (MRI) studies that showed neurodegenerative changes of the posterior cerebral cortex, only one study has assessed the functional aspects of visual brain function in HD using resting‐state functional MRI (Wolf et al., 2014). Here, functional connectivity changes were limited to the fusiform cortex in patients with HD, despite the presence of widespread posterior cortical atrophy. Still, little is known about brain function in patients with HD, and the link between structural cortical brain changes and functional impairments has not yet been fully investigated. Using different neuroimaging modalities and a visual cognitive test battery, this study aimed to improve the understanding of functional and structural alterations in the visual cortex in premanifest individuals (i.e., before the presence of motor symptoms) and in patients with early stage HD and investigate if there is an association between brain changes and visual cognitive task performance.

## 2 METHODS

### 2.1 Participants

A total of 59 participants (18 healthy controls, 20 premanifest HD gene carriers, and 20 premanifest HD gene carriers) were included in this cross‐sectional, observational study (Table 1).

**Table 1:** Demographic and clinical characteristics

	Controls	Premanifest HD	Manifest HD
*N*	18	21	20
Age, years	46.2 ± 10.7 (24.1–61.3)	37.4 ± 9.0* (23.2–53.0)	52.1 ± 10.8 (24.1–61.3)
Gender (male/female)	7/11	11/10	11/9
Handedness – Right (%)	15 (83.3%)	19 (90.5%)	15 (75.0%)
Education, years	17.0 ± 2.2	16.8 ± 3.2	16.4 ± 2.3
Current tobacco use	3 (16.7%)	4 (19.0%)	6 (30.0%)
CAG repeat length	N/A	41.8 ± 2.2 (38–45)	42.8 ± 2.4 (40–48)
UHDRS – TMS	1.8 ± 1.2 (0–5)	2.8 ± 1.0 (1–5)	27.2 ± 15.5** (8–52)
UHDRS – TFC	13 (11–13)	13 (10–13)	9 (6–13)**
Visual perception (compound Z‐score)	0.43 ± 0.43	0.34 ± 0.48	−0.61 ± 0.91**
Visual scanning and attention (compound Z‐score)	0.26 ± 0.43	0.13 ± 0.29	−0.26 ± 0.39**

Abbreviations: N/A = Not applicable; CAG = Cytosine, Adenine, Guanine; UHDRS‐TFC = Unified Huntington'’s Disease Rating Scale Total Functional Capacity; UHDRS‐TMS = Unified Huntington's Disease Rating Scale Total Motor Score.

Data are mean ± *SD* (range) for age, CAG repeat length, years of education, and UHDRS‐TMS. Numbers (%) are presented for handedness and tobacco use. Median (range) is given for UHDRS‐TFC. Compound standardized Z‐scores on visual cognitive tasks were calculated. Scaled Z‐scores were summed and averaged resulting in Z‐scores per visual cognitive domain. Mean Z‐scores ± *SD* per domain are presented. ANCOVA with group as simple contrast was used to assess differences in Z‐scores compared to controls, with age, gender, and years of education as covariates.

* Significant different compared to controls *p* < .05.

** Significant different compared to controls *p* < .001.

Individuals were recruited from the outpatient clinic of the Neurology department at the Leiden University Medical Center between January 2017 and September 2017. HD gene carriers required a positive genetic test with a cytosine, adenine, guanine (CAG) repeat expansion of 36 or more on the Huntingtin gene. Partners and HD gene‐negative relatives were recruited as healthy controls. All participants were between 18 and 65 years of age at the time of visit. To ensure an optimal primary visual acuity, individuals with an impaired primary visual ability (measured as below 0.5; i.e., 20/40 vision) on the visual acuity test or ophthalmic disorders were excluded from the study. Other exclusion criteria were additional major comorbidities not related to HD (including cardiovascular diseases, hypertension, diabetes mellitus, and other neurological disorders), the inability to undergo MRI scanning (due to metallic implants, claustrophobia, or pregnancy), or the participation in intervention trials. The Medical Ethical Committee of the Leiden University Medical Center approved this study, and written informed consent according to the Declaration of Helsinki was obtained from all participants.

### 2.2 Clinical assessments

Demographic information, CAG repeat length (for HD gene carriers only), and medical history was collected. Primary visual ability was measured with a visual acuity test. To assess the ability to perceive color differences the Ishihara Color Test was performed. Trained investigators assessed the degree of motor disturbances using the Unified Huntington's Disease Rating Scale ‐ Total Motor Score (UHDRS‐TMS), a scale that measures different domains that are characteristically impaired in HD, including the oculomotor function, the tongue protrusion, the gait and postural stability, and the presence of choreatic or dystonic movements (Huntington Study Group, 1996). Higher scores indicate increased motor impairment. Based on the UHDRS‐TMS, HD gene carriers were divided into 21 premanifest individuals, with a score of 5 or less, and 20 manifest individuals, with a score of more than 5 (Huntington Study Group, 1996). The UHDRS Total Functional Capacity (TFC) score was administered to assess five components of global daily functioning, including capacity to work, management of finances, ability to perform domestic chores, independency in activities of daily living (such as eating, bathing, and dressing), and care environment. Here, lower TFC scores indicate more impaired function. In manifest HD, the TFC score is used to divide participants into disease stages, in which Stages 1 and 2 represent an early disease stage and Stage 5 the most advanced stage (Shoulson & Fahn, 1979).

The cognitive battery used in this study consisted of specific neuropsychological assessments with a large visual component. All cognitive tasks were administered by certified neuropsychological investigators and lasted approximately 30 min.

The selection of cognitive assessments was based on findings from previous studies that focused on cognitive dysfunction in premanifest and manifest HD gene carriers (Lawrence, Watkins, Sahakian, Hodges, & Robbins, 2000; Lemiere et al., 2004; O'Rourke et al., 2011; Stout et al., 2012). The symbol digit modalities test (SDMT), stroop word reading test, and trail making test (TMT) were administered to assess visuospatial function, such as visual scanning and visual attention (O'Rourke et al., 2011; Smith, 1973; Stroop, 1935). Visual object perception was measured using subtests from the visual object and space perception battery and the Groningen intelligence test (Luteijn & Barelds, 2004; Warrington & James, 1991). Individual scores on each cognitive assessment were converted to standardized Z‐scores. These Z‐scores were summed and averaged resulting in cognitive domain scores to assess overall visuospatial and visual perceptual function.

### 2.3 Image acquisition

All participants underwent structural and functional MRI scanning on a 3.0 Tesla MRI scanner (Philips Achieva, Best, the Netherlands). Both structural and functional MRI data were acquired using a standard 32‐channel whole head coil. MRI proof glasses or lenses were used during scanning if participants had a visual acuity below 0.5 (20/40 vision) without correction, to ensure optimal primary visual ability during task performance. Anatomical three‐dimensional T1‐weighted images were acquired with the following parameters: TE = 3.3 ms, TR = 7.2 ms, flip angle = 9°, FOV = 256 × 240 × 176 mm and 176 slices with a slice thickness of 1 mm and no gap between slices, resulting in a voxel size of 1.00 × 1.00 × 1.00 mm, and scan duration of 9 min. All functional blood oxygen level‐dependent (BOLD)‐weighted echo‐planar imaging whole brain volumes were obtained with the following parameters: TE = 30 ms, TR = 3,000 ms, FOV = 212 × 198 × 158 mm, flip angle = 80°, and 48 slices with a slice thickness of 2.81 mm and slice gap of 0.5 mm, resulting in a voxel size of 3.31 × 3.31 × 2.81 mm, and scan duration of 5.42 min for the task fMRI, and 8 min for the resting state fMRI.

### 2.4 Image processing

#### 2.4.1 Structural MRI

Cortical morphology was examined using volumetric and thickness outcome measures. Gray matter density alterations of the visual cortex in premanifest and manifest HD gene carriers compared to controls were examined using VBM analysis, which involves a voxel‐wise comparison of the local concentration of gray matter between groups, including cortical surface area and cortical folding (Good et al., 2001). In addition, to investigate subtle brain changes, the cortical thickness of specific brain regions in the visual cortex were measured (Desikan et al., 2006).

#### 2.4.2 Voxel‐based morphometry

To assess structural voxel‐wise gray matter density differences of the visual cortex between groups, we used a standardized VBM analysis protocol (Good et al., 2001), using the FMRIB's Software Library (FSL, version 5.0.10, Oxford, United Kingdom) tools (Douaud et al., 2007; Smith et al., 2004). First, nonbrain tissue from all T1‐weighted images was removed using the semiautomated brain extraction tool (Smith, 2002). Then, these brain‐extracted images were segmented into different tissue types (i.e., gray matter, white matter, and cerebrospinal fluid). Quality control was performed on the brain extraction and gray matter segmentation images, and no data were excluded for further analyses. The gray matter images were aligned to the 2 mm Montreal Neurological Institute (MNI)‐152 standard space image, using nonlinear registration (Andersson, Jenkinson, & Smith, 2007; Jenkinson, Bannister, Brady, & Smith, 2002). An averaged study‐specific 4D template was subsequently created. Then, all native gray matter images were nonlinear registered to this study specific template and modulated to correct for local enlargements and contractions due to the nonlinear component of the spatial transformation (Good et al., 2001). Finally, the modulated gray matter images were smoothed with an isotropic Gaussian kernel with a sigma of 3 mm, which corresponds to a full width at half maximum (FWHM) smoothing kernel of 7 mm.

#### 2.4.3 Cortical thickness

Cortical thickness of specific regions of interest was measured using cortical parcellation implemented in FreeSurfer version 5.3.0 (Fischl & Dale, 2000). The FreeSurfer algorithm automatically parcellates the cortex and assigns a neuroanatomical label to each location on a cortical surface model, based on probabilistic information. Four occipital regions (lingual gyrus, pericalcarine cortex, cuneus, and lateral occipital cortex), one parietal region (superior parietal cortex), and three temporal regions (temporal pole, fusiform gyrus, inferior temporal gyrus) defined by the Desikan–Killiany atlas were selected for further analyses (Desikan et al., 2006), because these regions are all involved in the processing of visual stimuli (Prasad & Galetta, 2011). As there were no differences in cortical thickness between left and right hemisphere for any cortical region in our cohort, thickness was averaged across the two hemispheres for each region (see Supporting Information Table S1).

#### 2.4.4 Functional MRI

Alterations in brain function were assessed using task‐based and resting‐state fMRI. For the task‐based fMRI, changes in BOLD signal in response to a visual stimulus were calculated as a proxy for cortical neuronal *connectivity* (A. Dumas et al., 2012). In addition, brain function at rest was examined using resting‐state functional connectivity analysis with predefined visual resting‐state networks of interest (NOI; Beckmann, DeLuca, Devlin, & Smith, 2005).

#### 2.4.5 Task design

The visual stimulus for the task‐based fMRI scan consisted of seven blocks with an 8 Hz flashing black‐and‐white checkerboard pattern for 20 s, followed by 28 s of a gray screen with a red dot in the center of the screen. The visual stimuli were projected onto a screen, which the participant observed through a mirror attached to the head coil. To maintain attention and fixation to the checkerboard, each participant was asked to push on a button when the red dot position in the center of the screen changed from lighter to darker red, occurring at random intervals throughout the stimulus run. For the resting‐state fMRI measures, participants were asked to close their eyes, not to fall asleep, and not to speak during the complete scanning time.

#### 2.4.6 Preprocessing

All functional images were pre‐processed using the FEAT tool implemented in FSL (Smith et al., 2004). Pre‐processing steps included motion correction, (Jenkinson et al., 2002) spatial smoothing using a Gaussian kernel of 8 mm FWHM, high‐pass temporal filtering (with a cutoff of 48 s for task‐fMRI images, and 100 s for resting‐state fMRI images), and distortion correction of B0 inhomogeneity's. The preprocessed functional images were nonlinear registered to individual brain‐extracted T1‐weighted scans, which were registered to MNI‐152 standard space with boundary‐based‐registration (Jenkinson et al., 2002; Smith, 2002). No scans were excluded after **v**isual quality control to ensure correct registration.

#### 2.4.7 Vascular reactivity

Preprocessed task‐based functional MRI scans were further processed to measure cortical vascular reactivity in response to visual stimulation. For this analysis, BOLD timeseries had to be extracted from the preprocessed scans. To this end, a region of interest (ROI) was created for each participant based on the results of an initial subject‐level analysis. During this subject‐level analysis, brain regions were identified that reacted to the stimulus train using the general linear modeling approach implemented in FSL's FEAT (Smith et al., 2004). The degree to which each voxel responded to the stimulus train was expressed in a Z‐statistic map.

To obtain the functional ROI for each participant, a binary mask was constructed by taking the top 20% most activated voxels from this Z‐statistic activation map. Using this mask, an average BOLD timeseries was calculated for each participant by averaging across all masked voxels. The resulting timeseries was cut up into blocks that each contained stimulus period (20 s) and a subsequent rest period (28 s). Then, the timeseries of each block were expressed as percentage BOLD change using the mean value of all blocks. To minimize the effect of nonphysiologic noise on the block response, blocks with a percentage BOLD change greater than 3% were discarded.

Based on the method previously described by A. Dumas et al. (2012), a trapezoidal function was fit to the vascular reactivity response (i.e., percentage BOLD signal change) in the average BOLD timeseries, to describe the time‐to‐peak response, time‐to‐baseline and amplitude of the response. The time‐to‐peak was calculated from the beginning of the block at *t* = 0 to onset of the trapezoid ceiling. The time‐to‐baseline is defined as the duration from the end of the stimulus at *t* = 20 s to the baseline. Response amplitude was defined as the distance from baseline to the peak response. The entire algorithm described above was implemented in R (the R foundation for Statistical Computing, Vienna, Austria), version 3.4.2. The full source code, along with additional mathematical details, has been published on GitHub (https://github.com/jjhbw/TrapFit).

#### 2.4.8 Functional connectivity

After preprocessing of the functional resting state images, independent component analysis‐based automated removal of motion artifacts was used as a data‐driven method to identify and remove motion‐related independent components from our resting state fMRI data (Pruim et al., 2015). Then, functional connectivity analysis was performed using the dual regression method implemented in FSL, previously described by Filippini et al. (2009) and Hafkemeijer et al. (2015).

For the dual regression method, we used standardized resting state NOI to measure functional connectivity as BOLD signal changes in the brain in relation to similar alterations in predefined resting state networks (Beckmann et al., 2005). As we focused on the visual cortex, we used two templates of standardized resting state networks, namely the medial visual network (including the calcarine sulcus, precuneus, lateral geniculate nucleus, and primary visual cortex) and the lateral visual network (including the occipital pole, lateral occipital cortex, fusiform areas, and superior parietal regions; Beckmann et al., 2005). To account for noise, white matter and cerebrospinal fluid templates were also included in the analyses (Birn, 2012).

#### 2.4.9 Statistical analysis

Group differences in demographic and clinical outcome measures were analyzed using ANOVA, *χ* ^*2*^ test, and Kruskal–Wallis test when applicable for continuous, categorical and skewed data respectively. Analysis of Covariance (ANCOVA), with group as factor and a simple contrast, was used to analyze differences in cortical thickness, task‐based fMRI response parameters and cognitive task performance in premanifest and manifest HD compared to controls. Age, gender, and years of education were included as covariates. Multiple linear regression analyses in HD gene carriers (i.e., premanifest and manifest HD) were performed to assess the relationship between neuroimaging (dependent variable) and cognitive outcome measures (independent variable), adjusted for age, gender, years of education, and CAG repeat length. All independent variables were entered in one block. Separate linear regression models were used for each neuroimaging measure. An alpha‐level of <0.05 was used to determine significance. To account for multiple comparisons in this analysis, an adjusted *p* value was set based on the number of comparisons made for the analysis. Statistical analyses were performed using the Statistical Package for Social Sciences (SPSS for Mac, version 23, SPSS Inc.).

For the structural VBM and resting state functional connectivity data, statistical analyses to detect group differences between controls, and premanifest and manifest HD were performed using a general linear model in FSL with age and gender as covariates. FSL‐randomize was used for voxel‐wise nonpermutation testing with 5,000 permutations (Winkler, Ridgway, Webster, Smith, & Nichols, 2014). For the VBM analysis, a binary mask of the visual cortex extracted from the MNI‐152 standard space image was used. For the functional connectivity analysis, analyses of variance *F* tests were first performed to assess if the group averages accounted for a significant effect in each NOI. Then, two‐sample *t* tests were applied to obtain specific group differences (i.e., an increase or decrease) in functional connectivity. To account for the potential effects of local structural gray matter differences within and between the groups, individual gray matter density maps were used as additional voxel‐dependent covariate in the statistical design. The threshold‐free cluster enhancement (TFCE) technique was used to correct for multiple comparisons across voxels with family wise error (Smith & Nichols, 2009), with a significant *p* value of <0.05 as significant threshold. Brain structures that showed a significant difference between groups were identified on the TFCE‐statistic image using the Harvard–Oxford atlas and the cluster tool integrated in FSL.

## 3 RESULTS

### 3.1 Clinical characteristics

Demographic group characteristics are displayed in Table 1. Based on their functional capacity, manifest HD were in an early to moderate disease stage (4 patients in Stage 1, 15 patients in Stage 2, and 1 patient in Stage 3).

There were no significant group differences for gender, handedness, education level, and use of tobacco. CAG repeat length did not differ between premanifest and manifest HD. Premanifest HD were younger (F[2,56] = 10.90) compared to controls (*p* = .028) and manifest HD (*p* < .001). Furthermore, manifest HD had a higher UHDRS‐TMS compared to controls and premanifest HD (F[2,56] = 49.41, both *p* < .001) and a lower UHDRS‐TFC score compared to controls and premanifest HD (H[2] = 41.24, *p* < .001). On all visuospatial and visual perceptual tasks, manifest HD performed worse compared to controls. There were no significant differences in cognitive task performance between controls and premanifest HD.

### 3.2 Structure of the visual cortex

#### 3.2.1 Voxel‐based morphometry

**Figure 1:**
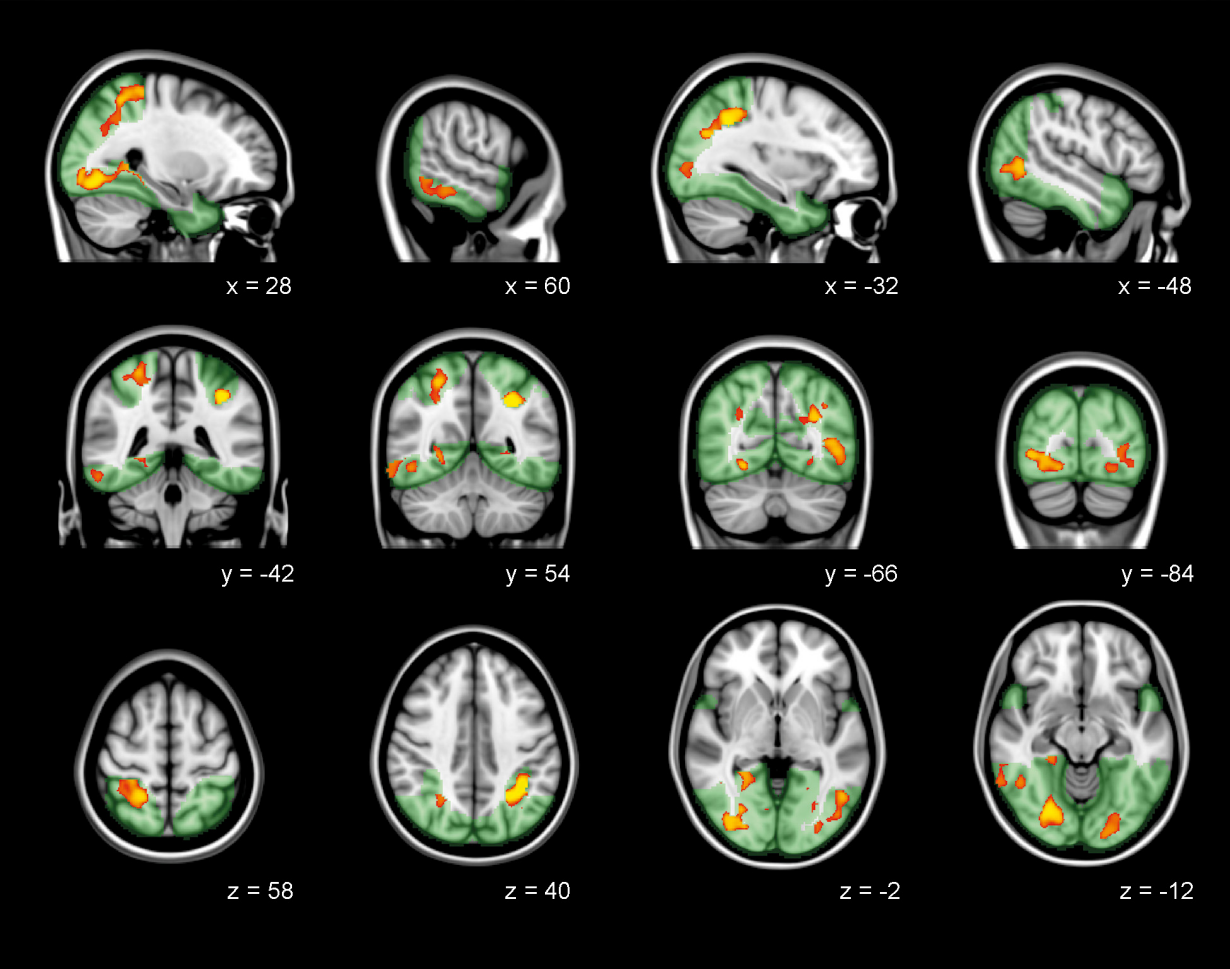
Gray matter volume loss in the visual cortex in manifest HD. VBM analysis. The significant family‐wise‐error corrected gray matter differences between manifest HD and controls (*p* < .05) are presented in red‐yellow, overlaid on sagittal, coronal, and transversal slices of Montreal‐neurological‐Institute‐152 standard T1‐weighted images. Green: Mask of visual cortex. Corresponding *x*, *y*, and *z* coordinates are given [Color figure can be viewed at http://wileyonlinelibrary.com]

Regional VBM analysis of the visual cortex was used to assess gray matter volume differences between groups. In manifest HD, significant cortical volume loss was identified bilateral in the fusiform gyrus, lingual gyrus, lateral occipital cortex and superior parietal cortex (Figure 1 and Table 2). Furthermore, the left occipital pole and right inferior temporal cortex showed volume loss in manifest HD compared to controls. No significant differences in gray matter volume were found between controls and premanifest HD.

**Table 2:** Gray matter volume loss in the visual cortex in manifest HD

		Side	MNI coordinates (mm)			*t* value	*p* value
Cluster voxel size	Anatomical regions		*x*	*y*	*z*		
870	Fusiform gyrus (BA 19/37)	R	24	−72	−10	4.78	.003
	Lateral occipital cortex (BA 19)	R	36	−80	−2	3.94	.004
	Lingual gyrus (BA 18)	R	26	−46	0	4.11	.014
741	Lateral occipital cortex (BA 19)	L	−46	−64	2	3.87	.012
	Occipital pole (BA 17)	L	−18	−92	−12	3.75	.022
	Fusiform gyrus (BA 19/37)	L	−26	−70	−6	3.32	.038
696	Superior parietal cortex (BA 7)	L	−36	−46	38	4.76	.012
606	Superior parietal cortex (BA 7)	R	22	−48	56	4.85	.007
398	Inferior temporal cortex (BA 20)	R	46	−48	−6	4.35	.019
15	Lingual gyrus (BA 18)	L	−26	−54	0	3.36	.046

Abbreviations: BA = Brodmann area; R = right hemisphere; L = left hemisphere.

Voxel‐wise identified regions of significant cortical volume loss in manifest HD compared to controls. All anatomical regions were identified using the Harvard–Oxford Subcortical and Cortical atlases and the cluster tool implemented in FSL. T‐statistics and corresponding *p* values are presented (with a TFCE‐family wise corrected *p* value of *p* < .05).

#### 3.2.2 Cortical thickness

To determine subtle cortical changes in the visual cortex, the cortical thickness of eight regions of interest (cuneus, fusiform gyrus, inferior temporal cortex, lateral occipital cortex, lingual gyrus, pericalcarine cortex, superior parietal cortex, and temporal pole) were additionally measured. Except for the pericalcarine cortex and temporal pole, significant cortical thinning was present in manifest HD in the cuneus, fusiform, and lingual gyri, and inferior temporal, lateral occipital and superior parietal cortices compared to controls (Table 3). Premanifest HD did not show any significant differences in cortical thickness for all regions of interest compared to controls.

**Table 3:** Cortical thickness in visual cortical regions

				Premanifest HD versus controls		Manifest HD versus controls	
	Controls	Premanifest HD	Manifest HD	Estimated difference [95% CI]	*p* value	Estimated difference [95% CI]	*p* value
Cuneus	1.95 ± 0.14	1.93 ± 0.13	1.82 ± 0.15	−0.05 [−0.14, 0.05]	0.344	−0.13 [−0.23, −0.04]	**.005**
Fusiform gyrus	2.71 ± 0.16	2.72 ± 0.11	2.55 ± 0.21	−0.03 [−0.15, 0.08]	0.539	−0.14 [−0.25, −0.04]	**.010**
Inferior temporal cortex	2.70 ± 0.13	2.73 ± 0.13	2.58 ± 0.18	−0.02 [−0.12, 0.09]	0.760	−0.10 [−0.20, −0.01]	**.039**
Lateral occipital cortex	2.11 ± 0.13	2.17 ± 0.11	1.99 ± 0.22	0.02 [−0.09, 0.13]	0.750	−0.11 [−0.21, 0.00]	**.048**
Lingual gyrus	2.10 ± 0.16	2.08 ± 0.09	1.96 ± 0.18	−0.07 [−0.17, 0.02]	0.143	−0.14 [−0.23, −0.05]	**.004**
Pericalcarine cortex	1.74 ± 0.16	1.71 ± 0.13	1.67 ± 0.16	−0.06 [−0.16, 0.05]	0.287	−0.06 [−0.16, 0.04]	.233
Superior parietal cortex	2.21 ± 0.16	2.19 ± 0.10	2.02 ± 0.19	−0.07 [−0.17, 0.03]	0.178	−0.18 [−0.27, −0.08]	**<.001**
Temporal pole	3.60 ± 0.36	3.77 ± 0.21	3.59 ± 0.40	0.15 [−0.08, 0.38]	0.206	0.02 [−0.21, 0.25]	.854

Data are mean ± *SD* (mm). Cortical regions from left and right hemispheres were averaged. Significant group differences are presented in bold. ANCOVA was used with group as simple contrast to assess group differences with age and gender as covariates.

Multiple linear regression analysis was performed to assess the relationship between cortical thickness and cognitive function in HD gene carriers (i.e., both premanifest and manifest HD). Besides the inferior temporal cortex, all regions showed significant associations with visual perceptual function, in which a decrease in cortical thickness was associated with worse cognitive function (Table 4). Visuospatial function was not related with changes in cortical thickness. The pericalcarine cortex and temporal pole were not included in these analyses as there were no significant group differences in cortical thickness.

**Table 4:** Associations between cortical thickness and visual cognitive task performance in HD gene carriers

	Cuneus				Fusiform gyrus				Inferior temporal cortex				Lateral occipital cortex				Lingual gyrus				Superior parietal cortex			
	*B*	*SE*	η^2^	*p*	*B*	*SE*	η^2^	*p*	*B*	*SE*	η^2^	*p*	*B*	*SE*	η^2^	*p*	*B*	*SE*	η^2^	*p*	*B*	*SE*	η^2^	*p*
Visual perception (compound Z‐score)	0.111	0.027	0.33	**<.001**	0.106	0.027	0.28	**<.001**	0.075	0.031	0.10	.020	0.130	0.030	0.39	**<.001**	0.097	0.022	0.24	**<.001**	0.130	0.027	0.57	**<.001**
Visual scanning and attention (compound Z‐score)	0.008	0.062	0.05	.893	0.061	0.060	0.01	.314	0.082	0.061	0.00	.185	0.022	0.069	0.04	.752	0.057	0.051	0.00	.275	0.008	0.065	0.03	.908

Unstandardized Beta (*B*), Standard Error (*SE*), and partial eta squared (η^2^) are presented adjusted for age, gender, years of education, and CAG. Unstandardized Beta represents change in cortical thickness (mm) for every 1‐point change in Z‐score for each cognitive domain. Significant *p* values (*p* < .004) are presented in bold.

The multiple linear regression analysis was subsequently performed in the manifest HD group. Significant associations were found between visual perceptual function and thickness of the fusiform gyrus (*B* = 0.136, *SE* = 0.036, *p* < .002) and lateral occipital cortex (*B* = 0.145, *SE* = 0.038, *p* < .002), corrected for multiple comparisons. There was no significant association between visuospatial function and cortical thickness.

### 3.3 Function of the visual cortex

#### 3.3.1 Vascular reactivity

**Figure 2:**
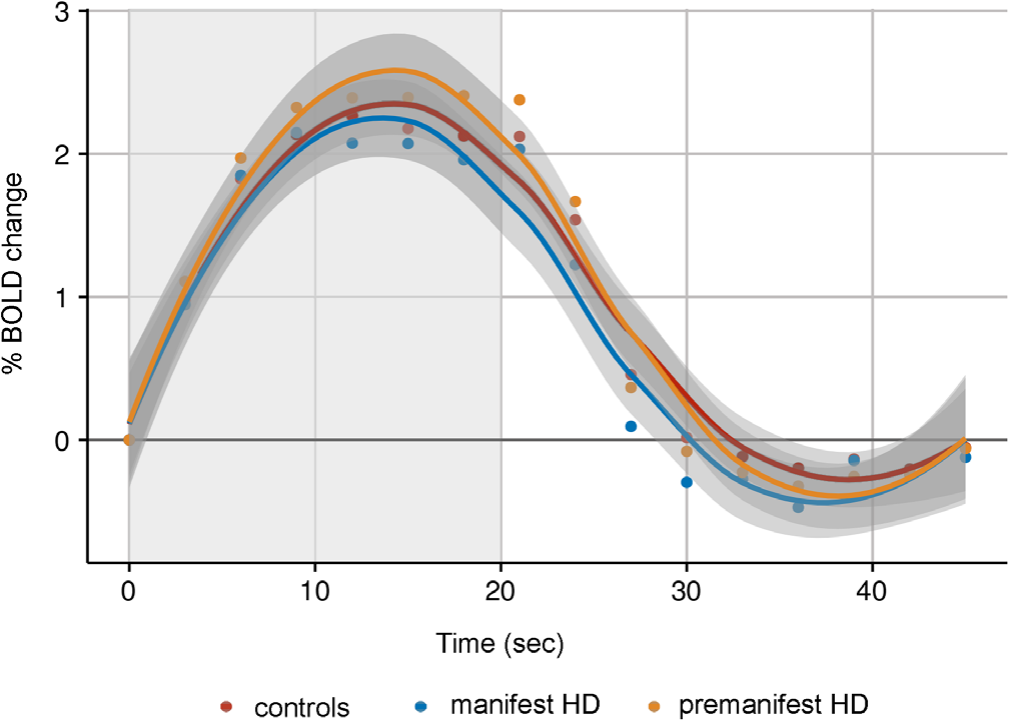
BOLD response to visual stimulation. Fitted average BOLD responses per group relative to baseline (0%). The gray area represents the duration of visual stimulation for 20 s [Color figure can be viewed at http://wileyonlinelibrary.com]

Vascular brain function of the visual cortex was examined by quantifying alterations in BOLD signal in response to visual stimulation. Relative to baseline (i.e., prior to the start of the stimulus), there were no significant group differences in changes of the average BOLD response for the time to peak, time to baseline and amplitude response (Figure 2).

#### 3.3.2 Functional connectivity

**Figure 3:**
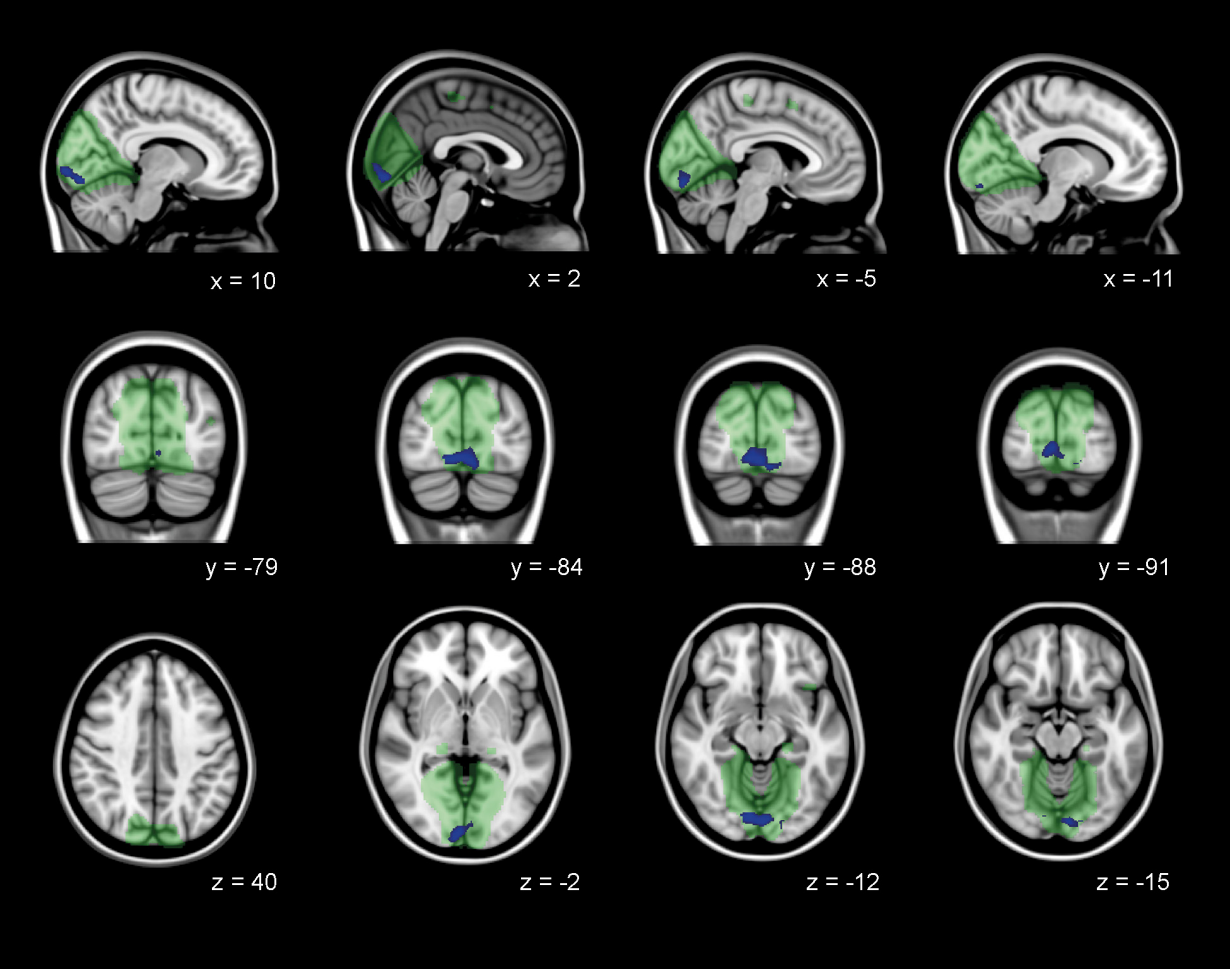
Decreased functional connectivity in medial visual network in manifest HD. Decreased functional connectivity independent of gray matter atrophy in manifest HD compared to controls in the medial visual network (green). Significant family‐wise‐error corrected regions are presented in blue overlaid on sagittal, coronal, and transversal slices of Montreal‐neurological‐Institue‐152 standard T1‐weighted images. Corresponding *x*, *y*, and *z* coordinates are given [Color figure can be viewed at http://wileyonlinelibrary.com]

Brain function at rest was assessed to detect disease specific functional connectivity network changes within the medial visual network and lateral visual network. Decreased functional connectivity between the bilateral lingual gyrus, occipital pole, and occipital fusiform gyrus and the medial visual network was present in manifest HD compared to controls, independent of local gray matter atrophy (Figure 3 and Table 5). No differences in functional connectivity with the medial visual network were observed between premanifest HD and controls. In addition, there were no group differences in lateral visual network functional connectivity.

**Table 5:** Decreased functional connectivity in medial visual network in manifest HD

	Side	MNI coordinates (mm)			*t* value	*p* value
Brain structure		*x*	*y*	*z*		
Lingual gyrus (BA 18) Occipital fusiform gyrus (BA 19/37)	R	12	−86	−14	4.65	0.013
Occipital pole (BA 17)	R	12	−98	−6	4.27	0.016
Lingual gyrus (BA 18) Occipital pole (BA 17)	L	0	−88	−14	4.43	0.015
Occipital fusiform gyrus (BA 19/37)	L	−14	−90	−16	3.39	0.035

Abbreviations: BA = brodmann area; R = right hemisphere; L = left hemisphere.

Brain structures in the medial visual network that showed reduced functional connectivity in manifest HD compared to controls, independent of physiological noise, age, and gender. Structures were identified using the Harvard–Oxford Cortical atlas and cluster tool implemented in FSL. T‐statistics and corresponding *p* values are presented (with a TFCE‐family wise corrected *p* value < .05). For each peak voxel *x*, *y*, and *z* coordinates in the MNI‐152 standard space image are given.

## 4 DISCUSSION

This study showed that changes in the visual cortex and visual cognitive deficits are present in early manifest HD gene carriers, but not in premanifest gene carriers.

The most pronounced volume loss and cortical thinning in manifest HD was found in the associative visual cortices, namely the lingual and fusiform gyri and lateral occipital cortex. Thinning of these cortical regions in the ventral occipital‐temporal pathway was associated with impaired visual perceptual function (i.e., object recognition tasks), suggesting that the neurodegenerative processes in the cortex might play a role in the visual deficits found in HD. Interestingly, the primary visual cortex (i.e., pericalcarine region and occipital pole) did not show neurodegenerative alterations and neuronal activity after visual stimulation also did not differ between groups, which suggest that basic visual processing remains preserved in early stages of the disease.

Our findings that cortical morphology of the primary visual cortex in early HD remained unaffected is in line with other studies that did not found atrophy of the primary visual cortex in both early and advanced disease stages (Johnson et al., 2015; Nana et al., 2014; Nopoulos et al., 2010). Still, our study is the first study that provides evidence of preserved basic visual processing function in early stages of HD using task‐based fMRI that involved a black‐and‐white checkerboard stimulus. The BOLD response to visual stimulation in the primary visual cortex was not different from controls in both manifest and premanifest HD, which suggests that incoming stimuli from the optic radiation and lateral geniculate nucleus in the thalamus are properly received and transmitted by the primary visual cortex to higher visual cortical areas.

In general, visual stimuli that are received by the primary visual cortex (V1) are then projected to the secondary visual cortex (V2), which plays a role in the color perception and orientation (Tootell, Tsao, & Vanduffel, 2003). Then, visual processing proceeds along the associative cortices, which can be divided into the ventral occipito‐temporal pathway (V4) involved in color processing and the recognition of objects and shapes, and the dorsal occipito‐parietal pathway (V3 and V5), involved in the processing of spatial information and movement perception (Kravitz, Saleem, Baker, Ungerleider, & Mishkin, 2013; Ungerleider & Haxby, 1994).

Visual scanning, attention, and visual object and shape recognition was measured using visuospatial and visual perceptual tasks respectively. Manifest HD gene carriers showed impairments in these domains, but only visual perceptual function was associated with cortical thickness. The SDMT and TMT were used to measure visuospatial function, as these tasks require visual scanning and attention skills (O'Rourke et al., 2011; Smith, 1973). However, these tasks also require a high motor demand and processing speed, which are known to be impaired in patients with HD. This might explain that no significant relationship was found between visuospatial task performance and thickness of the visual cortex.

The secondary visual cortex is located in the cuneus and lingual gyrus, which is involved in color discrimination and visual working memory (Tootell et al., 2003). From here, stimuli proceed towards the fusiform gyrus and lateral occipital cortex that are known to play a role in object and face recognition (Grill‐Spector, Kourtzi, & Kanwisher, 2001; Kanwisher, Mcdermott, & Chun, 1997). This supports our findings that an impaired visual perceptual function in HD was associated with reduced cortical thickness in these regions.

Other studies reported similar findings in early manifest HD, such as a reduced nerve cell number in the secondary visual cortex (Nana et al., 2014), volume loss of the occipital lobe (Tabrizi et al., 2009; Wolf et al., 2014), and thinning of the cuneus, lingual gyrus, and lateral occipital cortex that were associated with worse performance on cognitive tasks involving a visual component (Johnson et al., 2015; Rosas et al., 2008). Contrary to our findings, thinning and volume loss of the occipital lobe has also been observed in premanifest HD gene carriers (Johnson et al., 2015; Nopoulos et al., 2010; Rosas et al., 2005; Tabrizi et al., 2009). This process seems to occur in premanifest HD gene carriers that are within a decade or nearer to disease onset, suggesting a sudden increase in the rate of thinning around disease onset (Johnson et al., 2015; Nopoulos et al., 2010), but there are no longitudinal studies that can confirm this hypothesis. The difference with our findings might be explained by the fact that our cohort consisted of a heterogeneous, relatively young group of premanifest HD gene carriers with a median estimated time to disease onset of 16 years, based on the survival analysis of (Langbehn, Brinkman, Falush, Paulsen, & Hayden, 2004). In contrast, the multicenter TRACK‐HD and PREDICT‐HD studies included large cohorts of premanifest HD gene carriers which were divided into close (e.g., below 9 or 10 years) and far (above 10 to 15 years) from estimated disease onset. (Johnson et al., 2015; Nopoulos et al., 2010; Tabrizi et al., 2009) Only six premanifest participants in our study were within a decade or nearer to disease onset, which might explain the fact that we found no differences between controls and premanifest HD.

Function of the posterior cerebral cortex has been studied less extensively in HD. Our study examined brain function at rest, in addition to the assessment of brain function during task performance. Resting state fMRI can be used to study functional interactions between brain regions at rest (i.e., connectivity), and as no active input is required during resting state fMRI, the influence of the disease on task performance is not of concern (Biswal et al., 2010; Dumas, van den Bogaard, Hart, et al., 2013). Despite normal brain function after visual task stimulation, decreased functional connectivity at rest of the lingual and fusiform gyri, and occipital pole was found in manifest HD compared to controls within the medial visual network. One other study specifically focused on the visual cortex at rest using resting state fMRI in manifest HD and found only reduced connectivity in the left fusiform gyrus, despite widespread volume loss in the occipital cortex (Wolf et al., 2014). Our findings of reduced functional connectivity were also independent of gray matter atrophy in these regions, which might suggest that regional atrophy does not cause abnormal neural connectivity at rest.

To improve the understanding of functional alterations in different disease stages, we additionally examined brain function at rest in premanifest individuals. Compared to controls, we found no differences in functional connectivity for both medial and visual networks, which is contrary to a previous study that showed reduced connectivity in the right parietal and bilateral visual cortices of the medial visual network in premanifest HD gene carriers (Dumas, van den Bogaard, Hart, et al., 2013). Although using several methodological approaches, these reductions in whole brain functional connectivity in premanifest HD were not detectable over time in longitudinal studies (Odish et al., 2014; Seibert, Majid, Aron, Corey‐Bloom, & Brewer, 2012). A possible explanation for these discrepancies can be a selection bias, as participants with a faster rate of clinical decline might withdraw earlier from the study, leaving a relatively fitter premanifest group for longitudinal analyses (Odish et al., 2014).

Still, our study provides evidence of preserved brain function of the primary visual cortex at rest and after visual stimulation in manifest HD, but reduced function in the ventral occipito‐temporal pathway at rest. Structural alterations of the visual cortex seem, nevertheless, to be more pronounced and widespread than functional alterations in early manifest HD, even extending to the inferior temporal and superior parietal cortices. Together with previous studies that additionally found evidence of cortical thinning and volume loss in these regions in premanifest HD gene carriers that are within a decade to disease onset (Johnson et al., 2015; Nopoulos et al., 2010; Rosas et al., 2005; Tabrizi et al., 2009), this implies that structural alterations might precede functional alterations in HD. Future studies with larger sample sizes are, however, needed to examine brain function using tasks that involve other visual cognitive domains, such as object or facial emotion recognition, or visuomotor function.

A limitation of this study is that due to the cross‐sectional design and our heterogeneous group of premanifest HD gene carriers, it remains uncertain how the posterior cerebral cortex changes over time. It would be interesting to assess the progression of posterior cortical volume loss longitudinally and in addition measure the effect of volume loss on changes in neural connectivity. In this way, potential cortical biomarkers can be identified that can be used in future clinical trials. Another limitation of this study is the relative small sample size of our cohort, which additionally prevents examining the role of gender.

In conclusion, the ventral visual pathway, specifically the lingual and fusiform gyri and the lateral occipital cortex, showed most pronounced structural and functional alterations in early manifest HD. Our study is the first to provide evidence of preserved basic visual function in early disease stages after visual stimulation. Clinically, visual perceptual function was impaired and related to reduced cortical thickness of the ventral posterior brain regions. Still, changes in the visual cortex were not detectable in our premanifest HD group.

Our findings suggest that clinical visual deficits in HD are linked to atrophy of the posterior cerebral cortex, while basic visual function remains preserved in early disease stages.

## CONFLICT OF INTERESTS

E. M. Coppen, J. van der Grond, A. Hafkemeijer, and J. Barkey Wolf report no conflict of interests. R. A. C. Roos receives research grants from TEVA Pharmaceuticals and is advisor for UniQure.

## Supporting information


**Supplementary Table 1**. Cortical thickness per region in left and right hemispheres
