# Supplementary material for: Structural and functional changes of the visual cortex in early Huntington's disease
Source: Hum Brain Mapp. 2018 Aug 24;39(12):4776–86. doi: 10.1002/hbm.24322 (PMC6866293; doi:10.1002/hbm.24322)
Supplement: Supplementary file 1 — Supplementary Table 1. Cortical thickness per region in left and right hemispheres [file HBM-39-4776-s001.docx]

|  | **Controls** |  |  | **Premanifest HD** | |  | **Manifest HD** | |  |
| --- | --- | --- | --- | --- | --- | --- | --- | --- | --- |
|  | Left | Right | *p*-value | Left | Right | *p*-value | Left | Right | *p*-value |
| Cuneus | 1.96 ± 0.14 | 1.93 ± 0.15 | 0.177 | 1.93 ± 0.13 | 1.93 ± 0.14 | 0.734 | 1.82 ± 0.16 | 1.81 ± 0.16 | 0.532 |
| Fusiform gyrus | 2.72 ± 0.14 | 2.70 ± 0.19 | 0.413 | 2.74 ± 0.13 | 2.70 ± 0.12 | 0.220 | 2.59 ± 0.19 | 2.51 ± 0.24 | 0.312 |
| Inferior temporal | 2.69 ± 0.14 | 2.71 ± 0.15 | 0.472 | 2.74 ± 0.14 | 2.72 ± 0.14 | 0.400 | 2.58 ± 0.16 | 2.58 ± 0.21 | 0.889 |
| Lateral occipital cortex | 2.10 ± 0.13 | 2.12 ± 0.14 | 0.421 | 2.15 ± 0.10 | 2.18 ± 0.13 | 0.253 | 2.00 ± 0.23 | 1.99 ± 0.23 | 0.647 |
| Lingual gyrus | 2.09 ± 0.17 | 2.12 ± 0.16 | 0.213 | 2.15 ± 0.10 | 2.18 ± 0.13 | 0.895 | 1.97 ± 0.19 | 1.96 ± 0.17 | 0.634 |
| Pericalcarine cortex | 1.76 ± 0.19 | 1.72 ± 0.17 | 0.208 | 1.72 ± 0.13 | 1.71 ± 0.17 | 0.843 | 1.67 ± 0.17 | 1.68 ± 0.17 | 0.810 |
| Superior parietal cortex | 2.21 ± 0.15 | 2.21 ± 0.15 | 0.968 | 2.21 ± 0.11 | 2.17 ± 0.10 | 0.194 | 2.05 ± 0.18 | 2.00 ± 0.20 | 0.468 |
| Temporal pole | 3.56 ± 0.26 | 3.64 ± 0.52 | 0.439 | 3.76 ± 0.22 | 3.78 ± 0.28 | 0.763 | 3.62 ± 0.33 | 3.56 ± 0.53 | 0.500 |

**Supplementary Table 1.** Cortical thickness per region in left and right hemispheres

Mean cortical thickness and standard deviation in left and right hemispheres (in mm).

Paired t-test was used with significant p-value (two-tailed) < 0.05.
